# Supplement of High Protein-Enriched Diet Modulates the Diversity of Gut Microbiota in WT or PD-1H-Depleted Mice

**DOI:** 10.4014/jmb.2008.08003

**Published:** 2020-10-30

**Authors:** Yajun Xie, Ping Zhao, Zhigang Han, Wei Li, Dan Shi, Lei Xu, Qiying Yi

**Affiliations:** 1 The M.O.E. Key Laboratory of Laboratory Medical Diagnostics, The College of Laboratory Medicine, Chongqing Medical University, P.R. China; 2 State Key Laboratory of Silkworm Genome Biology, Southwest University, Chongqing, P.R. China; 3 Biological Science Research Center, Southwest University, Chongqing, P.R. China; 4 Laboratory Animal Center, Chongqing Medical University, Chongqing, P.R. China; 5 Key Laboratory of Molecular Biology for Infectious Diseases, Ministry of Education, Chongqing Medical University, Chongqing, P.R. China; 6 Fever Clinic, Dianjiang County Hospital of Traditional Chinese Medicine in Chongqing, Chongqing, P.R. China

**Keywords:** PD-1H, pupa, feed, gut microbiota, high-protein diet

## Abstract

Supplement of high-protein food plays an important role in improving the symptoms of malnutrition and the immune capacity of the body, but the association of high-protein diet and gut microbiota remained unaddressed. Here, we systematically analyzed the internal organs and gut microbiota in C57(WT) or PD-1H-depleted (KO) mice (T cells were activated) fed with pupae or feed for six weeks. We observed that the body weight gain in the mice fed with pupae increased less significantly than that of the feed group, while the villi and small intestine lengths in the pupa group were reduced compared with that of mice given feed. However, the average body weight of the KO mice increased compared with that of the WT mice fed with pupae or feed. Pupae increased the concentration of blood glucose in WT, but not in KO mice. Moreover, in the feed group, there was no difference in the weight of the internal organs between the WT and KO mice, but in the pupae-fed group, liver weight was decreased and spleen weight was increased compared with that of KO mice. The amounts/plural/amounts of Melainabacteria, Chloroflexi, and Armatimonadetes were specifically upregulated by pupae, and this upregulation was weakened or eliminated by PD-1H depletion. Some bacteria with high abundance in the feed-fed KO mice, such as Deferribacteres, Melainabacteria, Acidobacteria, *Bacteroidetes*, Spirochaetes and Verrucomicrobia, were decreased in pupae-fed KO mice, and Proteobacteria and *Deinococcus* were specifically enriched in pupae-fed KO mice. *Bacteroidetes*, Firmicutes and *Akkermansia* were associated with weight loss in the pupaefed group while *Lachnospiraceae* and *Anaerobiospirillum* were related glucose metabolism and energy consumption. Based on high-throughput sequencing, we discovered that some gut bacteria specifically regulated the metabolism of a high-protein diet, and PD-1H deficiency improved life quality and sustained blood glucose. Moreover, PD-1H responses to high-protein diet through modulating the type and quantity of gut bacteria. These findings provide evidence about the association among gut microbiota, T cell activation (for PD-1H depletion) and high-protein diet metabolism, have important theoretical significance for nutrition and health research.

## Introduction

A high-protein diet is beneficial for decreasing the risk of cardiovascular disease as well as glycemic control in patients with type 2 diabetes [1, 2], while long-term supplementation with a high-protein diet is effective in promoting weight loss in obese people while maintaining muscle mass [3, 4]. Feeding a high-protein diet to transgenic mice with sickle cell disease reduced both the frequency and severity of the histopathological changes associated with chronic organ injury [5]. It is well known that high protein status is intimately associated with the immune system [6], which is interrelated with mutiple aspects of physiological regulation, such as hormonal regulation, metabolic regulation, circadian rhythms, as well as nutrient utilization [7-10] . Although human studies linking diet, gut microbiota, and immunity are scarce, it is important to highlight the role of the gut microbiota in functioning innate immune response and its modulation by nutrition, and the subsequent alterations of body composition exerted by a high-protein diet.

High protein shifts the gut microbiota with the enrichment of some taxa, and in particular, the strain *Akkermansia muciniphila*, being correlated with reduction in fat mass, has been shown to be beneficial for reducing fat mass gain [11, 12]. While much evidence has demonstrated that microbial diversity is altered by dietary changes, much less is known about the impact of high-protein diet on the metabolic potential of gut microbiota and regulation of host immune system in diet-induced microbiota diversity. Programmed death-1 homolog (PD-1H), secondary to its immunoglobulin variable domain homology with PD-1, was shown to act as a co-inhibitory ligand on APCs that suppress T cell responses [13] . Experiments on PD-1^−/−^ mice indicate that PD-1H expressed on CD4+ T cells suppresses acute inflammation and enhances antitumor immunity [14]. PD-1H deficiency strengthened the immunity and altered the composition of gut microbiota asssociated with resistance to DSS-induced colitis in our previous work (unpublished data). This evidence suggested that mice lacking certain components of the immune system have altered gut microbiota that can be transmissible between mice and change susceptibility to intestinal inflammation [15, 16] . PD-1^−/−^ mice exhibited alteration in their gut microbiota caused by impaired ability of T follicular helper (TFH) cells, which results in dysregulated selection of proper IgA precursor cells in the absence of PD-1 [17] . Pupae as a food source are high in protein, but there is scant evidence for the association of gut microbiota, high-protein foods (*e.g.*, pupae) and immune responses.

Here, we systematically analyzed the internal organs and gut microbiota in WT or PD-1H KO mice (T cells were activated) fed with pupae or feed for a long period. Analysis using 16S rRNA indicated that the intestinal microbiota of the pupa-fed group were mainly composed of *Bacteroidetes*, Firmicutes and *Akkermansia*. *Lachnospiraceae* and *Anaerobiospirillum* have been demonstrated to be associated with glucose metabolism and energy consumption. Melainabacteria, Chloroflexi, and Armatimonadetes were specifically upregulated by pupae, and their upregulation was weakened or eliminated by depletion of PD-1H. PD-1H expression or a high-protein diet of pupae were involved in altering gut microbial communities and provided novel evidence about the association among gut microbiota, T cell activation (for PD-1H depletion) and high-protein diet metabolism.

## Materials and Methods

### Mice Group Administration

Male C57BL/6J mice (WT) weighing 10-13 g (approximately 3 weeks old) were purchased from the Laboratory Animal Center of Chongqing Medical University (CMU). The nutrient compositions of the silkworm pupae and feed diets were measured at Sichuan Academy of Agricultural Sciences in China. PD-1H KO mice were kindly donated by Dr. Deng (The First Affiliated Hospital, Sun Yat-sen University). PD-1H homozygote (+/+) C57BL/6J mice generated from PD-1H heterozygotes were bred and maintained in conditions identical to those of PD-1H–knockout (KO) mice and used as controls (WT) for PD-1H KO studies. All the mice were kept in the Laboratory Animal Center of CMU in specific pathogen-free (SPF) conditions with a 12-h light-dark cycle. This study was approved by the Ethics Committee of CMU (Ref. No. 2018020) and performed at the Laboratory Animal Center of CMU [SYXK(YU)2018-0003]. Twenty-four mice were numbered and equally divided into 4 groups: (1) WT-F group (Feed-fed mice), (2) WT-P group (Pupa-fed mice), (3) PD-1H KO-F group (Feed-fed mice), and (4) PD-1H KO-P group (Pupa-fed mice).

### Sample Collection and Organ Coefficient Measurement

The initial body weight of each mouse at 3 weeks of age in each group was similar. The body weight was measured once per day at 9:00 AM throughout the experimental period. On the last day of the experiment, the mice were anesthetized in a relatively sealed space. All the following procedures were performed under sterile conditions. The abdomen was opened and the gut was a septically removed and immediately placed on an ice-cold plate. The cecum contents were collected. Briefly, the cecum of each mouse was cut with surgical scissors and the contents of the cecum were aspirated into the EP tube using a syringe without a needle. Then, the colon was gently washed with sterile saline to remove its contents. Samples were simultaneously vortexed and subjected to continuous ultrasonic processing for 3 min. Next, the samples were left in an ice-cold water bath for 30 min and then centrifuged at 5,000 ×g for 15 min at 4°C. The supernatant was transferred into a fresh tube. The colon length was measured using a ruler and the remaining colon tissues were placed into the EP tube to be immediately frozen and stored at -80°C until analysis. Finally, organs such as the heart, spleen, lung, kidney, and liver were collected and weighed.

### Histological Analysis

Colon tissues were fixed in 4% paraformaldehyde for at least 30 min and then embedded in paraffin wax to maintain their natural shape and tissue architecture during long-term storage. The tissues were cut into sections as thin as 4 to 5 μm with a microtome. The sections were then stained with hematoxylin and eosin (HE) and examined at 100× magnification using a high magnification optical microscope (Leica Aperio AT2, Germany).

### DNA Isolation from Colonic Contents

The metagenomic DNA in the colon contents of the mice was extracted according to the manufacturer’s instructions using the QIAamp DNA Stool Mini Kit (Qiagen, Germany). DNA integrity and size were verified using 1.0% agarose gel electrophoresis. The purity and concentration of the DNA were measured using the NanoDrop 2000 spectrophotometer (Thermo, USA). The 16S ribosomal DNA (rDNA) gene was analyzed (*n* = 6 per group) to evaluate the bacterial diversity by using Illumina HiSeq (Novogene Bioinformatics Technology Co., Ltd.).

### 16S rDNA Sequencing and Bioinformatics Analysis

The enteric microorganisms in the fecal and cecal samples were measured using a metagenomics method. Microbial genomic DNA was obtained using a Fast DNASpin Kit for Soil (MP Biomedical). The V4 region of the 16S rDNA was amplified with the 515F-806R primers specific for the V4 hypervariable regions (5’-GTGCCAGCMGCCGCGGTAA-3’ and 5’-GGACTACHVGGGTWTCTAAT-3’, respectively). The products were purified and quantified using Gene Clean Turbo (MP Biomedical) and the Quant-iT PicoGreen dsDNA Assay Kit (Life Technologies), respectively. Libraries were prepared using TruSeq DNA LT Sample Preparation Kits (Illumina) and sequenced on an Illumina MiSeq platform according to the manufacturer’s recommendations provided by Beijing Novogene Genomics Technology Co., Ltd. (China). The raw sequences were screened. The short lengths (< 200 bp) were then removed, and the paired-end reads with mismatch-free, overlapping sequences longer than 10 bp were assembled according to their sequence similarity. Paired-end reads were assigned to samples based on their unique barcode, which was cut off along with primer sequences before further analyses. Quality filtering on the raw tags was performed under specific filtering conditions to obtain high-quality clean tags according to the QIIME (V1.7.0) [18]. Sequence analyses were performed by Uparse software (Uparse v7.0.1001) [19]. Sequences with ≥ 97% similarity were assigned to the same OTUs. The sequences were then clustered into OTUs based on 97% identity using QIIME 2 [20]. The representative sequences for each OTU were aligned to identify the species using PyNASTin QIIME [21]. OTU abundance information was normalized using a standard sequence number corresponding to the sample with the least number of sequences. Rare faction curves for alpha diversity were generated to assess the efficiency of the sequencing depth and to represent and compare microbial communities. Species richness was estimated using Chao1. The beta diversity of the microbial communities was determined by visual assessment using principal coordinate analysis (PCoA) plots. Similarity analysis was based on weighted UniFrac distances (QIIME) and was calculated according to a one-way non-parametric multivariate analysis of variance.

### Statistical Analysis

The data are presented as the means ± SEM with respect to the number of samples (n) in each group and analyzed using GraphPad Prism 5 and Origin 8.5. The differences between samples were analyzed by one-way analysis of variance(ANOVA) with Duncan’s multiple range test. The results were considered significant when *p* < 0.05. Statistical significance between multiple treatment groups was determined by ANOVA and Student *t*-test.

## Results

### Pupae Diet Is Rich with High-Protein, Low-Fat Contents

**Fig. 1:**
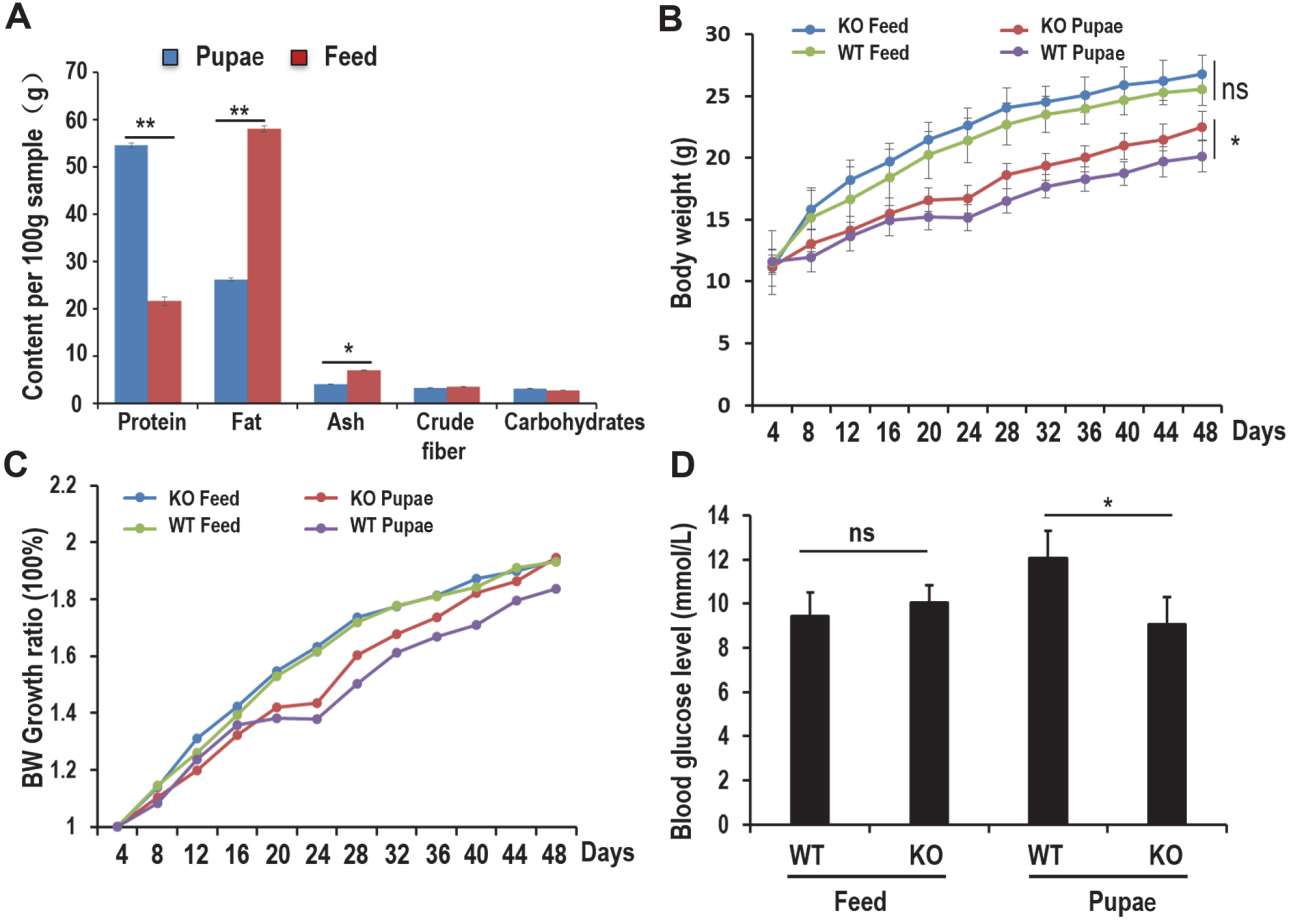
Different effect of nutritional composition between pupae and feed on biological properties. (**A**) Comparision of the nutritional composition between pupa and feed. All values represent means ± SEM, **p < 0.01. (**B**) Body weight gain (g) and (**C**) body weight growth ratio. Mice were fed with pupae or feed after initiation of experiments at 3 weeks of age, and weighed once every two days until the age of 10 weeks. Each point with errors represents the mean body weight ± SEM, *p < 0.05. (**D**) Fasting blood glucose levels (mmol/l) (*n* = 6 in feed-fed group, *n* = 6 in pupa-fed group) All values represent means ± SEM, *p < 0.05, **p < 0.01.

The nutrient compositions of both the silkworm pupae and feed diets were measured and compared (Fig. 1A). The protein content of silkworm pupae on a dry weight basis was around 54%, which is higher than the 21.6% of the feed. The fat content of the pupae was about 26% (dry weight), which is lower than the 58% of the feed (Fig. 1A). The ash, crude fiber, and carbohydrate contents of the pupae (dry weight) were also assayed, and the results were similar to those of the regular feed (Fig. 1A). The high-protein, low-fat content of pupae as a food source holds enormous nutrient potential for the human diet and animal feed.

### Pupae Feeding Suppressed the Growth of WT and PD-1H KO Mice

The body weight gain in WT and PD-1H KO mice fed with pupae for 48 days increased less significantly than that of the feed group (27.2 ± 0.14 vs. 25.8 ± 0.17, *p* < 0.05) (Figs. 1B and 1C). Although PD-1H mediated suppression of autoimmunity, there was no significant effect on the body weight between KO and WT mice statistically, but the average body weight of the PD-1H KO mice was higher than that of WT. The blood glucose levels for WT fed a pupa-based diet were much higher than those of the WT mice fed with feed (12.0833 ± 0.93112 vs 9.45 ± 1.0472) (Fig. 1D), but the differences in other groups were not statistically significant.

### Pupae Feeding Decreased the Small Intestine Length and Villus Number in the Colon

**Fig. 2:**
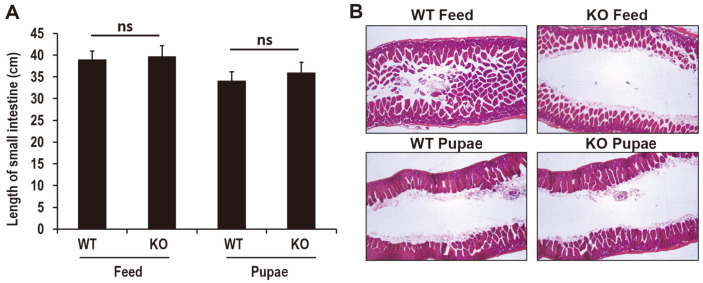
Influence of long-term intake of pupae on intestinal length and morphological alterations. (**A**) Intestinal length (*n* = 6 in feed-fed and pupa-fed group respectively). ns represents no significance. (**B**) Representative photo micrographs of H&E intestinal sections (*n* = 4 mice per group), Scar bar = 100 μm.

**Fig. 3:**
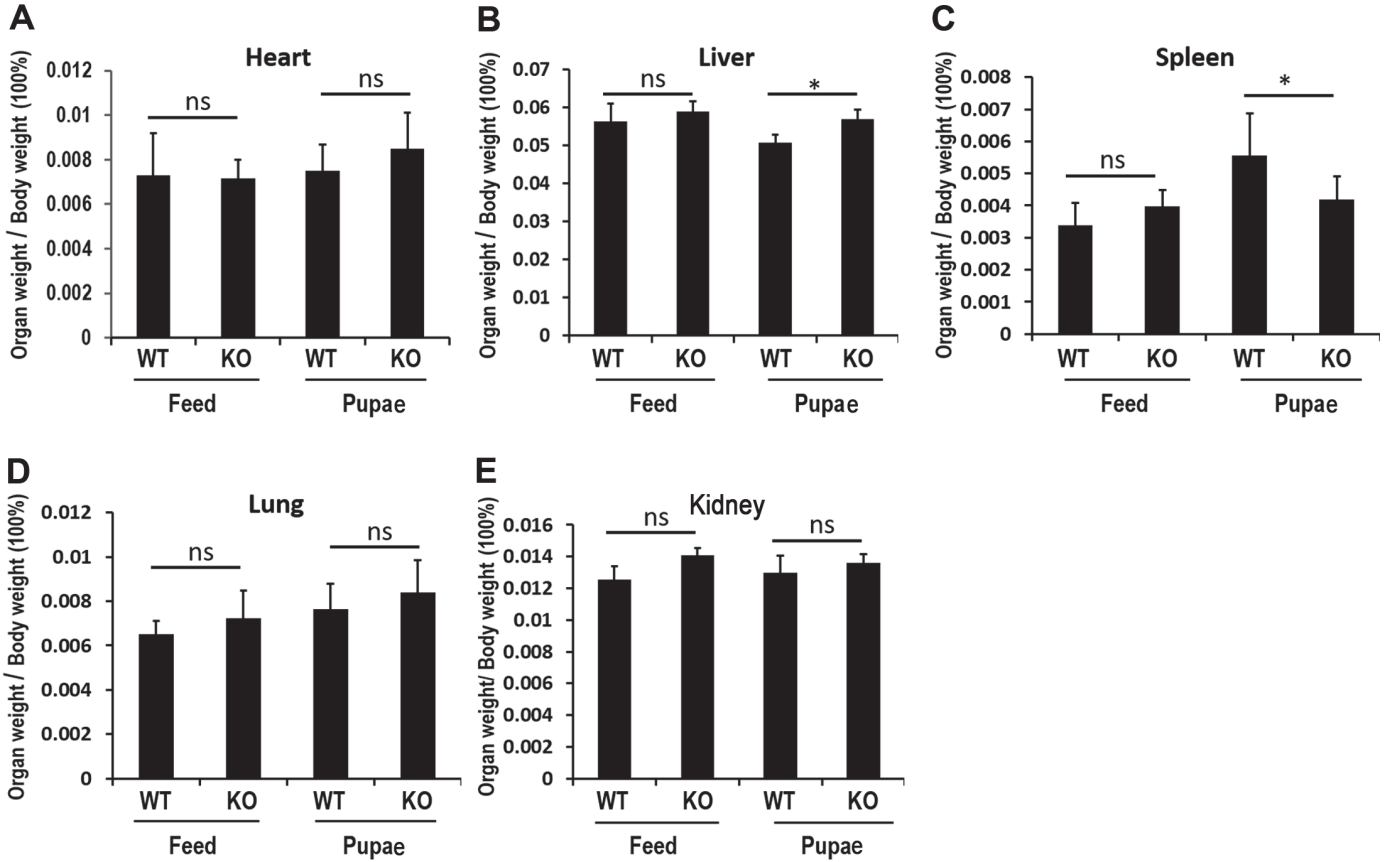
Relative organ weight analysis of WT and PD1H KO mice fed with pupae or feed. (**A**) The relative heart weight. (**B**) The relative liver weight. (**C**) The relative spleen weight. (**D**) The relative lung weight. (**E**) The relative kidney weight. Wet weight of five organs was measured and normalized to whole body weight. All values represent means ± SEM, *<0.05, **p < 0.01.

The small intestine length of WT (39 ± 1.949) or KO (39.7 ± 2.47) mice fed with pupae was significantly reduced compared with that of mice fed with feed (34.1 ± 2.1 or 35.94 ± 2.35) (Fig. 2A). After high-throughput scanning of pathological sections with the Leica Aperio AT2 system, the sections were observed under 10× magnification (Fig. 2B). The four-layer structure of the colon wall was clear. The lamina propria and submucosa were thick and the villi were intact in the feed group compared with the pupae group in the WT mice. The tunica muscularis mucosa of the feed group was visible. However, the villi in the pupae group were shorter than those of the feed group among the WT or KO mice. The organs (including heart, spleen, lung, kidney, and liver) were further weighed, but there was no difference between the WT or KO groups fed with pupae or feed (Fig. 3). Nevertheless, the liver index in the pupa-fed group decreased significantly compared with the feed-fed group of WT and this decrease was reversed in PD-1H KO mice (0.056315 ± 0.04579 vs. 0.050739 ± 0.00197).

### Diversity of the Bacterial Community during the Treatment Period

**Fig. 4:**
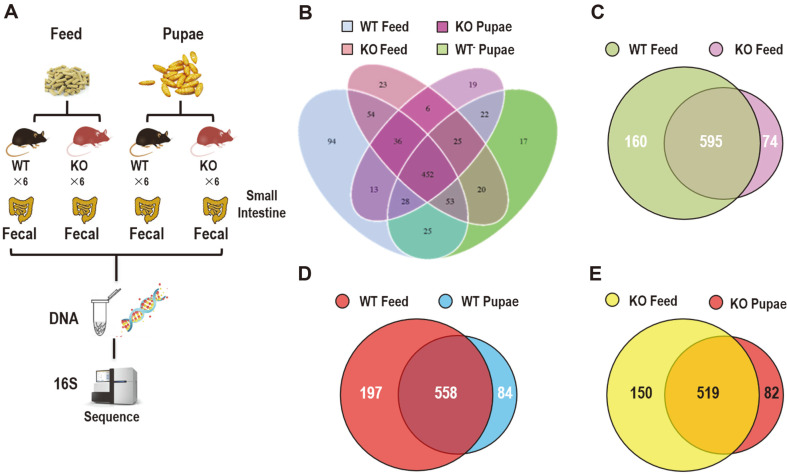
16S rDNA sequencing strategy and Venn diagrams displaying the distribution of unique and shared OTUs in different groups. (**A**) Outline of microbial genomic DNA sequencing procedure and data evaluation of enteric microorganisms in the fecal samples between pupa and feed groups. (**B**) Venn diagram of gut bacterial community showing degree of overlapping among 4 groups by OTU analysis. (**C**) Venn plot showing degree of overlapping bacterial species between WT and KO groups fed with feed by OTU analysis. (**D**) Venn plot showing degree of overlapping bacterial species between WT mice fed with pupae or feed by OTU analysis. (**E**) Venn plot showing degree of overlapping bacterial species between KO mice fed with pupae or feed by OTU analysis. Note: Values in core represent the bacterial species detected in corresponding individual group (indicated by different color).

**Fig. 5:**
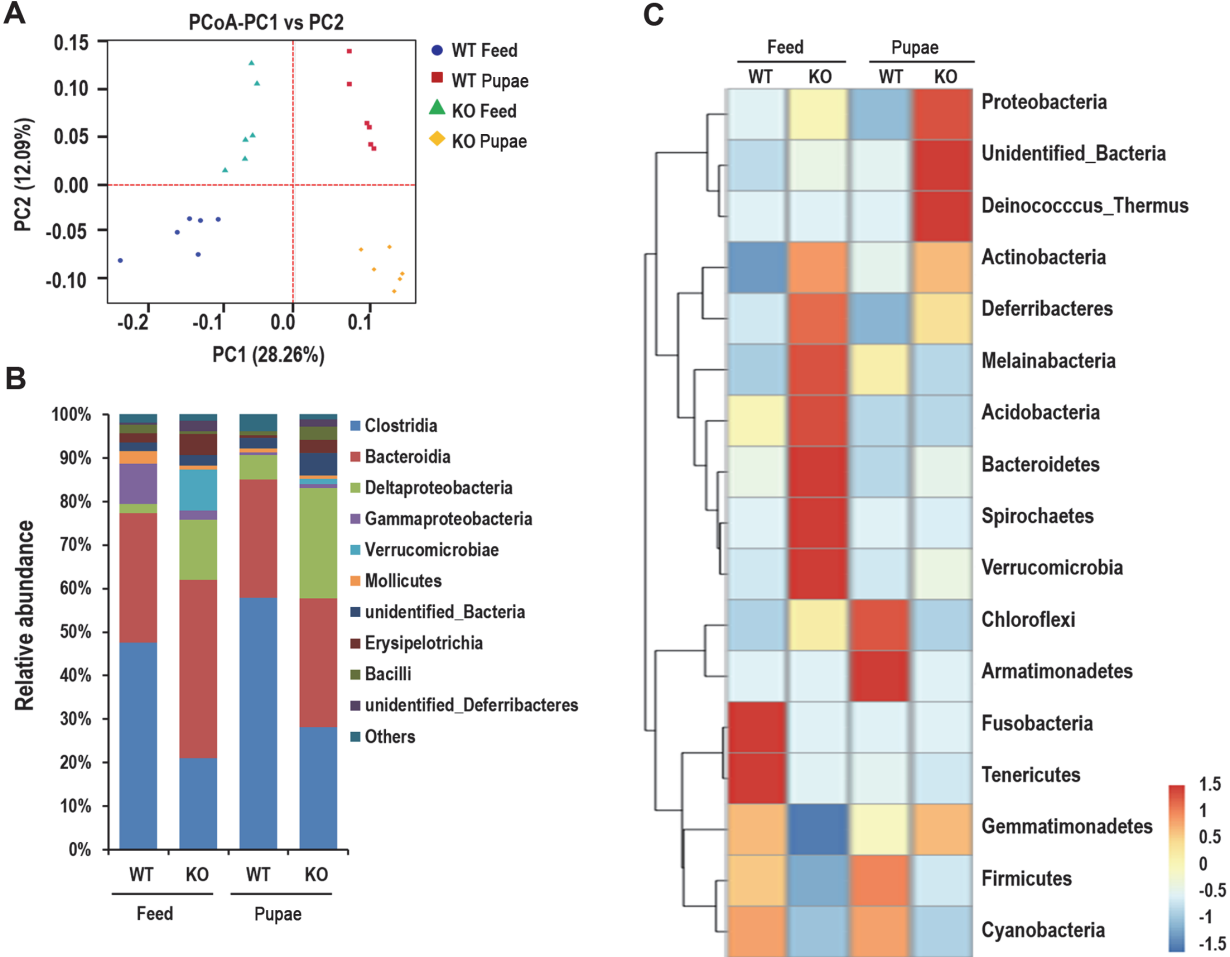
Changes in gut bacterial community proportional membership between different mice fed with pupae or feed. (**A**) Principal coordinate analysis (PCA) of 16S sequences from 24 samples using unweighted UniFrac distances of fecal microbiota among the 4 groups, which show distinct separation of samples based on their diets into feed- and pupa-fed groups in WT and PD-1H KO mice. (**B**) Relative read abundance of different bacterial classes within different communities. Only the top 10 enriched class categories are shown in the figure. The color-coded bar plot shows the average bacterial class. Sequences that cannot be classified into any known group are assigned as “Other bacteria”. (**C**) Hierarchical clustering and heatmap depicting relative abundance of taxa summarized to phylum level across different groups of interest for the top 14 OTUs. Relative abundance of each taxa within each sample is represented by color on the heatmap, with red and blue indicating the highest and lowest abundance, respectively.

Using the Illumina HiSeq 2500 platform, 144,218 good pyrosequencing reads were obtained from 24 samples. After discarding sequences that had no near-neighbors in the entire Greengenes database, 143,957 reads were delineated into 3451 OTUs at the 97% similarity level with distance-based OTU and richness. The raw reads of 24 libraries were submitted to the SRA database of NCBI (Accession Number: PRJNA600299). The experimental workflow combined 16S rRNA gene sequencing and metabolite profiling to examine the changes in the gut microbiome of mice fed either pupae or feed (Fig. 4A). Briefly, DNA was isolated from fecal pellets, amplified by polymerase chain reaction (PCR) using 16S rRNA–specific primers followed by 150 × 150 bp paired-end sequencing using the Illumina MiSeq platform. The resultant sequencing reads were processed using the QIIME and Metastats software packages to reveal gut microbiome changes in mice fed pupae or feed. There were many remarkable overlaps in differentially abundant OTU between compartments (Figs. 4B-4E). The OTUs enriched in the mice fed with pupae appeared successfully in feed-fed groups, as 452 out of the 755 or 669 OTUs were enriched in the WT or KO communities fed a regular diet (Fig. 4A). The feed-fed WT and KO mice share 595 of the 829 OTUs. Moreover, 160 and 74 OTUs were mainly in the feed-fed WT and KO groups, respectively (Fig. 4C). The WT mice fed with feed and pupa share 558 of the 839 OTUs, 197 and 84 OTUs were mainly found in the WT group fed with feed and pupa, respectively (Fig. 4D). In addition, 150 and 82 OTUs were differentially abundant in the KO group feeding with feed and pupa, and 519 were shared in these two groups (Fig. 4E). Unconstrained PcoAs of unweighted UniFrac distances were performed to visualize and compare the differences of significant separation between microbial communities. PcoA of the samples showed significant separation by 4 groups using unweighted UniFrac (ANOSIM P = 0.001) (Fig. 5A). Principal coordinate1 (PC1) (percent variation explained: 28.26%) of the unweighted UniFrac separated all 4 sets of samples, while PC2 (12.09%) separated the pupa feeding groups from other samples even further.

### Structure and Composition of Gut Microbiota

Total DNA from the fecal pellets was sequenced at the V4 region of 16S rDNA gene to investigate the difference in the intestinal microbiota in WT or KO mice fed with pupae or feed. The dominant classes in the 4 groups were Clostridia, Bacteroidia, and Deltaproteobacteria. The relative abundance of Clostridia and Deltaproteobacteria in WT-F and PD-1H KO-F groups was reduced compared with WT-P and PD-1H KO-P groups. However, the relative abundance of Bacteroidia in the PD-1H KO-F group was greatly increased compared to that of the PD-1H KO-P group (Fig. 5B). Comparisons of the relative abundance at the phylum level in the heatmap showed that the WT-P group had a higher abundance of Actinobacteria, *Bacteroidetes*, Proteobacteria, Verrucomicrobia, Deferribacteres, Acidobacteria, and Melainabacteria than WT-F group (Fig. 5C). In particular, the abundance of Firmicutes, Fusobacteria, and Chloroflexi was lower in WT-P mice than the WT-F group (Fig. 5C). Interestingly, higher proportions of Actinobacteria, Proteobacteria, such as *Deinococcus-Thermus*, and *Bacteroidetes* were observed in the PD-1H KO-P than in the PD-1H KO-F group.

### Variation in Microbial Communities among Different Diets in Mice

**Fig. 6:**
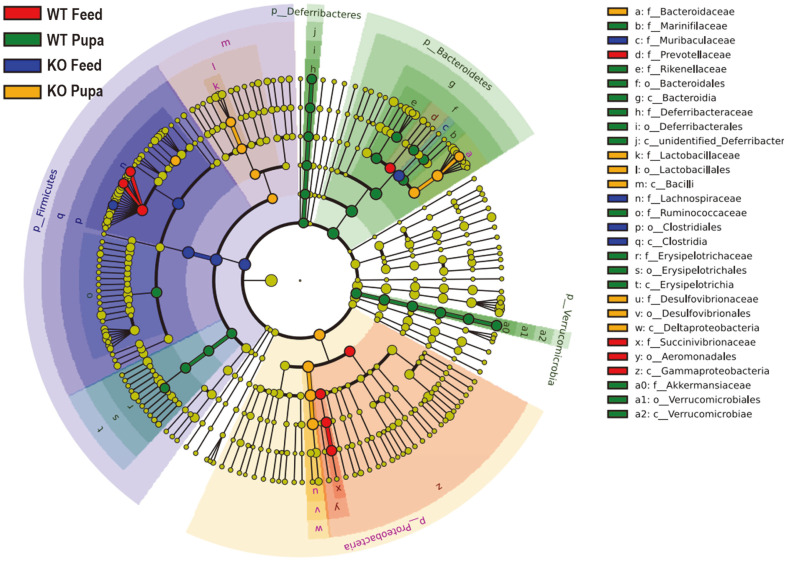
Comparing microbial variations of LEfSe analysis in different groups at the family level. Linear discriminant analysis (LDA) effect size taxonomic cladogram comparing bacterial communities in four groups: colors correspond to the individual group (red indicating WT fed with feed, green indicating WT fed with pupae, blue indicating PD- 1H KO mice fed with feed and yellow indicating PD-1H KO mice fed with pupae). Significantly discriminant taxon nodes are colored and branch areas are shaded according to the highest-ranked variety for that taxon. For each taxon detected, the corresponding node in the taxonomic cladogram is colored according to the highest-ranked group for that taxon. Each circle’s diameter is proportional to the taxon’s abundance. If the taxon is not significantly differentially represented between sample groups, the corresponding node is colored yellow. Highly abundant and select taxa are indicated. Seven rings of the cladogram stand for domain (innermost), phylum, class, order, family, genus, and species (outermost), respectively. Enlarged circles in color are the differentially abundant taxa identified to be metagenomic biomarkers.

Linear discriminant analysis (LDA) effect size (LEfSe) modeling was employed to analyze the pattern of the intestinal microbiota further. The resulting significant taxa were used to generate a taxonomic cladogram illustrating the different groups. There were significant differences in the community compositions among the 4 groups as reflected by the cladogram drawn at the family level. *Bacteroides*, Deltaproteobacteria, Verrucomicrobia, and *Akkermansia* were abundant in the WT-P compared with the WT-F at the family level, while *Lachnospiraceae*, Firmicutes, and Clostridia were the dominant microbiota in the WT-F at the phylum level (Fig. 6). *Gammaproteobacteria* and Aeromonadales were further identified as critical microbiota in the PD-1H KO-F compared with the WT-F group at the order level.

### Prediction of Microbial Functions

**Fig. 7:**
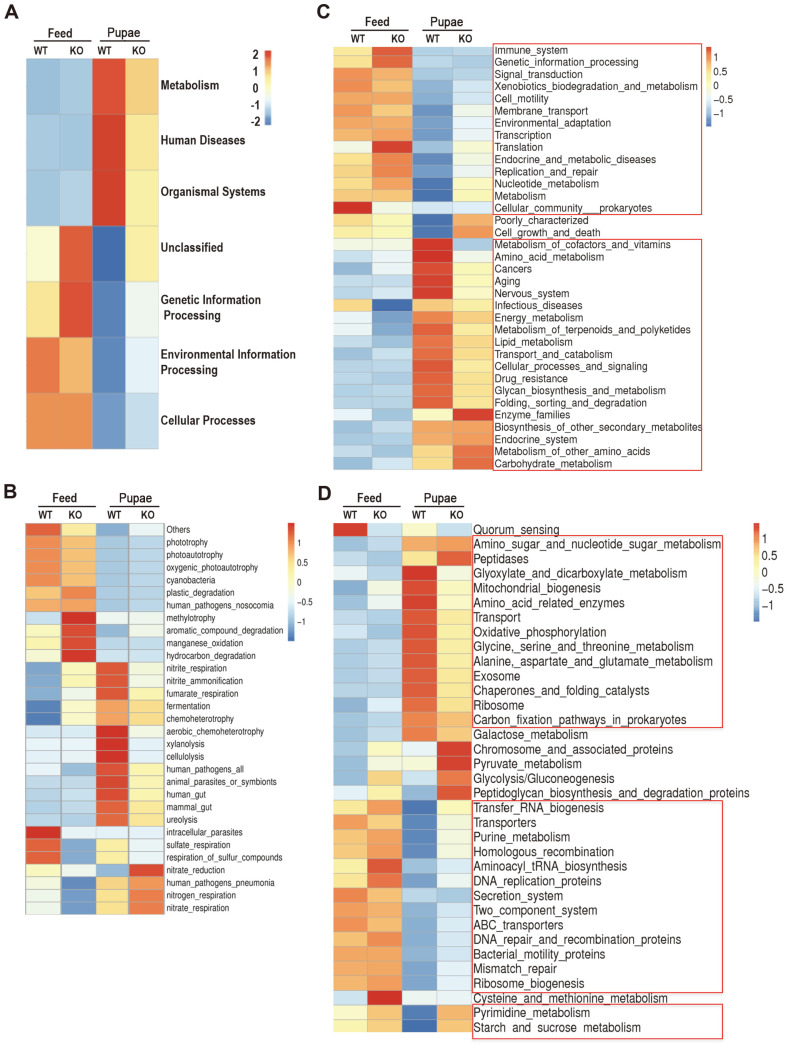
Predicted microbial functions that vary significantly at different groups using Tax4fun analysis. Heat map and hierarchical clustering were used to compare abundance of predicted KEGG categories at level 1 (**A**), level 2 (**C**), and level 3 (**D**). Relative abundances of KOs involved in the KEGG pathways associated with gut microbiota at genus level (**B**). Significant associations are indicated using colored blocks. Heatmaps show distribution of Log2 fold changes in gene expression between two groups. Red and blue colors on the column squares represent high and low patterns for some genes between two groups, respectively.

Tax4fun was used to predict the functional profiles of gut microbiota in WT or PD1H KO mice administered with feed or pupae. Significant differences in KEGG pathways were analyzed with statistical analysis of taxonomic and functional profiles (STAMP). There was a higher proportion of KEGG pathways assigned to metabolism, human diseases, and organismal systems in the WT and PD1H KO mice fed with pupae than the feed groups at the first level (Fig. 7A). The overrepresented KOs in the feed groups compared with the pupa-fed groups included functions for carbohydrate and cholesterol metabolic processes such asmethyl-accepting chemotaxis protein (K03406), ATP-binding cassette (K06147) and putative ABC transport system permease protein (K02004), and for membrane transport such as putative ABC transport system ATP-binding protein (K02003), multiple sugar transport system permease proteins (K02004, K02025, K02027) and a ferrous iron transport protein (K04759). Furthermore, the abundance of genes such as carbamoyl-phosphate synthase large subunit (K01955), glutamine synthetase (K01915), β-glucosidase (K05349), β-galactosidase (K01190) and hexosaminidase (K12373) related to amino acid and glucolipids metabolism were highly enriched in the pupa-fed groups compared with feeds (Fig. 7B). A total of 35 genes in the feed and pupa groups were in the KEGG level 2 category. Most belonged to amino acid metabolism, carbohydrate metabolism, and lipid metabolism (Fig. 7C). In the KEGG level 3 subcategories, pupa-fed WT mice had a higher proportion assigned to the amino acid metabolism (Ala, Asp, Glu, Gly, Ser, and Thr). The genes related to glycolysis/gluconeogenesis metabolism, peptidoglycan biosynthesis and degradation, and carbon fixation pathways had a higher abundance in the pupa-fed PD-1H KO mice (Fig. 7D).

## Discussion

The gut microbiota participates in several essential metabolic functions in a high-protein diet that contributes to host health [22]. It has been reported that dietary patterns are closely related to distinct bacterial communities in the human gut [23]. Animal studies have provided evidence that dietary components influence the structure and functionality of the intestinal microbiota [24, 25]. The protein content in the pupa-based diet was much higher than that of the feed. Among the top 10 genera, the abundance of *Lachnospiraceae* and *Anaerobiospirillum* of the pupa group at the family level was significantly lower than that of the feed-fed group. Reportedly, *Lachnospiraceae* and *Anaerobiospirillum* positively correlated with glucose metabolism and energy consumption [26-28].

*Bacteroides* and *Akkermansia*, increased in the gut microbiota by ingestion of pupa proteins, are known for their glycan, protein degrading and fat metabolism [29, 30]. Furthermore, it is indicated that increasing the intestinal abundance of *Akkermansia* can protect against obesity-linked metabolic syndrome and contribute to beneficial metabolic effects [31, 32]. The long-term consumption of a high-fat or high-protein diet has been reported to reshape gut microbiota, particularly by increasing the proportion of Firmicutes in relation to *Bacteroidetes* [33, 34]. It was reported that 4 weeks of high-protein diet could result in an increase in branched-chain fatty acids, a decrease in butyrate, and a decrease in Roseburia/Eubacterium numbers [35].

Concerning the predicted function, microbiota in the pupa group significantly differed in terms of predicted functional level of KEGG pathways compared with the feed group in WT mice. These data suggest that *Lachnospiraceae* and *Anaerobiospirillum* in the pupa group upregulate the metabolic process partially through different microbiota functions as identified by the second level of KEGG, such as amino acid-related enzymes, energy metabolism, lipid metabolism, glycan biosynthesis, and degradation. The corresponding pathways of pupa-fed WT and PD-1H KO mice were both upregulated in the amino acid metabolism (Ala, Asp, Glu, Gly, Ser, and Thr) at the third level. Most of these pathways were closely related with a higher content of protein in pupa feeding than feed in mice. However, some KEGG pathways related to transcription, replication, and repair and nucleotide metabolism were downregulated at the second level. The corresponding pathways at the third KEGG level were transfer-RNA biogenesis, purine metabolism, aminoacyl tRNAbio synthesis, DNA replication proteins, and mismatch repair, biological roles of which are related to DNA integrity and stability of gene expression. The relative abundances of the inferred microbial functions might be related to the long-term pupa intake in this study. For instance, microbial genes related to amino acids and glucolipids metabolism were more abundant in the microorganisms in the pupa group than in the feed because they participated in the digestion of more protein and fatty acids. Interestingly, the KEGG pathway genes in the pupa-fed groups associated with endocrine and metabolic diseases, such as diabetes mellitus and cardiovascular disease, were upregulated. It is speculated that by-products of silkworm pupae farming are not only an excellent nutritional food source but also a weight loss-boosting remedy.

The PD-1H KO mice exhibited an increased frequency of activated T cell response. We analyzed the possible correlations between PD-1H and *Anaerobiospirillum*, *Bacteroidetes* and *Lachnospiraceae* in KEGG pathways to determine whether the stimulated T cells were linked to the gut microbiome. There are subtle differences among the intestinal microbiota obtained from PD-1H KO and WT mice. Our results showed that *Desulfovibrionaceae* and *Lachnospiraceae* levels of 16S rRNA in PD-1H KO mice were higher at the family level compared to WT mice administered with the same feed. The high-protein/low-fat content of pupa associated with decreasing the abundance of *Lachnospiraceae* and *Anaerobiospirillum*, which helps to supply more nutrients and energy to the host.

Although the composition and function of gut microbiota are closely related to the immune system and metabolic function of the host, the effect of the high-protein content of pupa on the human microbiota composition has only been studied to a minor extent. Diet can change blood glucose concentration, and the pupa is a high-protein food. Maintaining a healthy blood glucose level is critical for the prevention and control of metabolic syndromes [36]. Our results showed that the blood glucose concentration in pupa-fed WT mice was higher than that of feed-fed WT mice. A high-protein/fat ratio in the diet protected against high-fat diet-induced obesity, hepatic lipid accumulation, and a significant reduction in survival. To what extent high-protein or fat intake modulates energy expenditure via the gut microbiome, linking protein-dependent changes in the gut microbiota with metabolism, remains to be solved. Nevertheless, to what extent such differences between proteins in pupa reflect direct metabolic effects in the host or to what extent the microbiota plays a causal role, need further explanation.

## Conclusions

The study demonstrated that different nutritional levels of pupae had significant effects on the microbial communities and metabolic functions in two murine models. Furthermore, the dominant microbiota and physiological responses in mice correlated with the nutritional content in pupae or feed. Despite the comprehensive analyses provided by the present study, there is a profound need for more in-depth investigations into the correlation between the microbiota composition and the nutritional profile of a pupa-based diet.

## Supplemental Materials



Supplementary data for this paper are available on-line only at http://jmb.or.kr.
