# Supplementary material for: Supplement of High Protein-Enriched Diet Modulates the Diversity of Gut Microbiota in WT or PD-1H-Depleted Mice
Source: J Microbiol Biotechnol. 2020 Oct 30;31(2):207–16. doi: 10.4014/jmb.2008.08003 (PMC9705843; doi:10.4014/jmb.2008.08003)
Supplement: Supplementary file 1 [file jmb-31-2-207-supple.pdf]

J. Microbiol. Biotechnol.
https://doi.org/10.4014/jmb.2008.08003

KO_Hierarchy	C57.PUP	C57.F	PD1H.F	PD1H.PUP
Metabolism	0.4676544	0.43705047	0.43791	0.45851577
Human_Disea	0.02997951	0.02683163	0.02679932	0.02857262
Environmenta	0.11585691	0.13720976	0.13437997	0.12294799
Organismal_S	0.0210068	0.01854361	0.01863846	0.01977789
Genetic_Infor	0.23967289	0.24285835	0.24441452	0.24141469
Unclassified	0.05376816	0.05423652	0.05469293	0.05438046
Cellular_Proce	0.07206133	0.08326966	0.08316479	0.07439058

KO_Hierarchy	C57.PUP	C57.F	PD1H.F	PD1H.PUP
Glycan_biosyr	0.04617396	0.02925519	0.02975181	0.04021148
Amino_acid_n	0.0907788	0.08530638	0.0859764	0.08627596
Nucleotide_m	0.04229516	0.04397159	0.04425903	0.04350944
Metabolism	0.01470932	0.01609911	0.01613605	0.01577482
Lipid_metabo	0.02557195	0.02378383	0.02355518	0.02502448
Infectious_dis	0.00660903	0.00660512	0.00650238	0.00659333
Translation	0.09641881	0.09663379	0.09715791	0.09668418
Cellular_proce	0.01676014	0.01424357	0.01426741	0.01562645
Aging	0.00400049	0.00304321	0.00304158	0.00341872
Cellular_comr	0.01769099	0.01878358	0.01788564	0.01774145
Immune_syste	0.00301486	0.00309145	0.00314249	0.00301821
Poorly_charac	0.00952223	0.01023209	0.01009958	0.01033221
Membrane_tr	0.08681958	0.10387486	0.10155306	0.09373469
Nervous_syste	0.0035112	0.00284478	0.00289051	0.00307367
Environmenta	0.00134343	0.00178705	0.00178379	0.00149389
Signal_transdu	0.02744184	0.03256798	0.03208007	0.02771763
Enzyme_famil	0.02380346	0.0235345	0.02330794	0.02450953
Endocrine_and	0.00290789	0.00307875	0.00312783	0.00300756
Metabolism_c	0.03346496	0.03227032	0.03226365	0.0318581
Biosynthesis_c	0.01514054	0.01348223	0.01369821	0.01518144
Drug_resistan	0.01207365	0.00963615	0.00953754	0.01116234
Transport_anc	0.03081286	0.02513512	0.02585389	0.02910488
Energy_metab	0.04467847	0.04358497	0.04304902	0.04437097
Folding,_sortir	0.02791763	0.0253502	0.02535857	0.02694886
Metabolism_c	0.01482745	0.014008	0.01387425	0.01520013
Genetic_inforr	0.01193536	0.01288329	0.01341542	0.01187348
Cancers	0.00557691	0.00494666	0.00511262	0.00526326
Metabolism_c	0.0107676	0.00992607	0.00958785	0.01032292
Replication_ar	0.0974506	0.10137991	0.10228929	0.09924299
Carbohydrate_	0.11222444	0.10831097	0.10923263	0.11377498
Xenobiotics_b	0.00792761	0.00961643	0.00935403	0.00827633
Endocrine_sys	0.00783112	0.00738573	0.00740224	0.00785043
Cell_growth_a	0.00876666	0.00902596	0.00899906	0.00912023
Cell_motility	0.01479083	0.030325	0.0304262	0.01842403
Transcription	0.01788585	0.01949444	0.01960874	0.01853867

KO_Hierarchy	C57.PUP	C57.F	PD1H.F	PD1H.PUP
Pyrimidine_m	0.01894047	0.01922536	0.01937575	0.01939243
Exosome	0.01726332	0.01509813	0.01522535	0.01642225
Chromosome	0.01241713	0.01228144	0.0125604	0.01297885
Purine_metab	0.02335469	0.02474623	0.02488328	0.02411701
Mitochondria	0.01270906	0.01251033	0.01257991	0.01260308
Pyruvate_met	0.01352879	0.01339907	0.01352228	0.01377005
Aminoacyl_tR	0.01492032	0.01506396	0.01517953	0.01496592
Carbon_fixatio	0.011214	0.00947944	0.00962272	0.0109193
DNA_replicati	0.01362151	0.01434962	0.01465926	0.01396309
Mismatch_rep	0.00941382	0.01082596	0.01083919	0.00984921
Starch_and_su	0.01104864	0.01227579	0.01257147	0.01261568
Amino_acid_r	0.01938103	0.01844193	0.01866857	0.01882825
Amino_sugar_	0.01392541	0.01222884	0.0124077	0.01402846
Homologous_	0.00943181	0.01012505	0.01021987	0.00981504
Secretion_syst	0.0087609	0.00995483	0.00974417	0.00869009
Glycolysis/Glu	0.0129957	0.0129485	0.01316885	0.01323756
Two_compon	0.02203794	0.02905686	0.02859078	0.02359197
Glycine,_serin	0.00911564	0.00772517	0.00782245	0.00848286
Peptidoglycar	0.00959542	0.00967799	0.00980731	0.00997298
Ribosome_bic	0.01224531	0.01295373	0.01300318	0.01247046
Chaperones_a	0.01083527	0.0093505	0.00934345	0.01023493
Ribosome	0.01589014	0.01510576	0.01528232	0.01564974
Transport	0.0090529	0.00575289	0.0055739	0.00753485
Transporters	0.05804139	0.06841671	0.06700652	0.06321186
Quorum_sens	0.01179138	0.01226276	0.01158046	0.01157362
Transfer_RNA	0.0245752	0.02522649	0.0254098	0.02511887
Cysteine_and_	0.00935534	0.00931793	0.00963182	0.00935158
Peptidases	0.01715038	0.01631724	0.01618583	0.01770698
Oxidative_pho	0.00932017	0.00766779	0.00742111	0.00845075
Alanine,_aspa	0.01304158	0.01171281	0.0118053	0.01245264
Glyoxylate_an	0.0089715	0.00790205	0.00769625	0.00801975
DNA_repair_a	0.03322156	0.03431684	0.03449328	0.03351883
Bacterial_mot	0.00663894	0.0138055	0.01383182	0.00814643
Galactose_me	0.00998873	0.0086966	0.00884154	0.00972775
ABC_transpor	0.013276	0.01828003	0.01772801	0.01453914

KO_Hierarchy	C57.PUP	C57.F	PD1H.F	PD1H.PUP
K02337	0.00281205	0.00261882	0.00265887	0.00275882
K02470	0.00204566	0.00257439	0.00267341	0.00219512
K03406	0.00398554	0.01006165	0.01015957	0.00545434
K00986	0.00113095	0.00251506	0.00262836	0.00123839
K02026	0.00112934	0.00442496	0.00430932	0.00193485
K07497	0.00302399	0.0020777	0.00243816	0.00298688
K01190	0.0061289	0.00319956	0.00324488	0.00525589
K02025	0.00126437	0.00493264	0.0048223	0.00215229
K01915	0.0029627	0.00264593	0.0026773	0.00277496
K03046	0.00326425	0.00286304	0.00289139	0.00313279
K02027	0.00133289	0.00464033	0.00445134	0.00209862
K03798	0.00188708	0.00311285	0.00307353	0.00220169
K02004	0.00522723	0.00681651	0.00733988	0.00659617
K02003	0.00268013	0.00372971	0.00401625	0.0032622
K12373	0.00467405	0.00058746	0.00078769	0.00312176
K05349	0.00402102	0.00276956	0.00298024	0.00486887
K04759	0.00227819	0.00291123	0.00305502	0.00249908
K02529	0.0018402	0.00383465	0.00387015	0.00250942
K01153	0.00295014	0.00267686	0.00292472	0.00312707
K03296	0.00785067	0.00183897	0.00177872	0.00497618
K03763	0.00114361	0.00297634	0.00300089	0.00166445
K09687	0.00175791	0.00341644	0.00367953	0.00229552
K06147	0.00665989	0.01182486	0.01203083	0.00944712
K03088	0.00470262	0.00258006	0.00283965	0.00422134
K02014	0.01092086	0.00248818	0.00226426	0.00970912
K03657	0.0032747	0.00458185	0.00448758	0.00356596
K02469	0.00242966	0.00307852	0.00316546	0.00263115
K01955	0.00429568	0.00323081	0.00326114	0.00372773
K02355	0.00285538	0.0030511	0.00307103	0.00307181
K03043	0.00301215	0.0029171	0.00294103	0.00294584
K03701	0.00520184	0.00327964	0.00327643	0.00391423
K03737	0.00298987	0.00259948	0.00288832	0.00299541
K01952	0.00245977	0.0026744	0.00275546	0.00261256
K00936	0.00251701	0.00253165	0.00244902	0.00260374
K03654	0.00260058	0.00107212	0.00104992	0.00203765
